# Differential Volatile Signatures from Skin, Naevi and Melanoma: A Novel Approach to Detect a Pathological Process

**DOI:** 10.1371/journal.pone.0013813

**Published:** 2010-11-04

**Authors:** Tatjana Abaffy, Robert Duncan, Daniel D. Riemer, Olaf Tietje, George Elgart, Clara Milikowski, R. Anthony DeFazio

**Affiliations:** 1 Molecular and Cellular Pharmacology, Miller School of Medicine, University of Miami, Miami, Florida, United States of America; 2 Division of Biostatistics, Department of Epidemiology and Public Health, Miller School of Medicine, University of Miami, Miami, Florida, United States of America; 3 Marine and Atmospheric Chemistry, Rosenstiel School of Marine and Atmospheric Science, University of Miami, Miami, Florida, United States of America; 4 Systaim GmbH, Zurich, Switzerland; 5 Department of Cutaneous Surgery and Dermatology, University of Miami, Miami, Florida, United States of America; 6 Department of Pathology, University of Miami, Miami, Florida, United States of America; 7 Department of Neurology, University of Miami, Miami, Florida, United States of America; City of Hope National Medical Center, United States of America

## Abstract

**Background:**

Early detection of melanoma is of great importance to reduce mortality. Discovering new melanoma biomarkers would improve early detection and diagnosis. Here, we present a novel approach to detect volatile compounds from skin.

**Methods and Findings:**

We used Head Space Solid Phase Micro-Extraction (HS-SPME) and gas chromatography/mass spectrometry (GC/MS) to identify volatile signatures from melanoma, naevi and skin samples. We hypothesized that the metabolic state of tissue alters the profile of volatile compounds. Volatiles released from fresh biopsy tissue of melanoma and benign naevus were compared based on their difference in frequency distribution and their expression level. We also analyzed volatile profiles from frozen tissue, including skin and melanoma.

**Conclusions:**

Three volatiles, 4-methyl decane, dodecane and undecane were preferentially expressed in both fresh and frozen melanoma, indicating that they are candidate biomarkers. Twelve candidate biomarkers evaluated by fuzzy logic analysis of frozen samples distinguished melanoma from skin with 89% sensitivity and 90% specificity. Our results demonstrate proof-of-principle that there is differential expression of volatiles in melanoma. Our volatile metabolomic approach will lead to a better understanding of melanoma and can enable development of new diagnostic and treatment strategies based on altered metabolism.

## Introduction

Although the idea that changes in tissue are an indication of disease dates back to the ancient Greeks [1], full understanding of many pathological processes at the molecular system level is still lacking. In the current post-genomic era, we are faced with an enormous amount of data from genomic, transcriptomic, and proteomic studies. Systems biology approaches are being used to integrate these large data sets in an attempt to understand how macromolecular networks may control phenotype. Metabolomics, the study of small molecule metabolites is now emerging. The metabolome is defined as the complete set of metabolites, low molecular weight intermediates which are context dependent, varying according to the physiological, developmental or pathological state of the cell, tissue, organ or organism [2]. The advantage of metabolomics is that metabolites are downstream of both transcription and translation, at the endpoint of the “omics” cascade and thus may be most predictive of phenotype [3], [4]. Because of the chemical diversity of metabolites, no single platform for their detection can be used. Currently used platforms include NMR spectroscopy, LC/MS, GC/MS and MS The study of the metabolome can be divided into two classes: 1. metabolic profiling, which relates to the study of a group of metabolites specific for a particular metabolic pathway; and 2. metabolic fingerprinting, which compares patterns (i.e. fingerprints) of metabolites that change in response to disease [5].

In this paper, we studied skin, naevi and melanoma volatile metabolomes; their volatile metabolomic fingerprints or signatures. Our hypothesis is that melanoma tissue emits unique volatile compounds which are either direct metabolic products of melanoma patho-physiology or epiphenomena. In order to study volatile metabolic compounds, we used gas chromatography/mass spectrometry (GC/MS), a powerful method for studying volatile organic compounds. First, we collected volatiles by using the rapid and solvent free Head-Space Solid Phase Micro-Extraction method (HS-SPME) initially developed by J. Pawliszyn [6]. Volatiles from the collection fiber were easily desorbed into a gas chromatograph, separated on a non-polar column, and detected by a mass spectrometer. The identity of each compound was determined by comparison with a mass-spectral database. Analysis of volatile components from fresh melanoma and naevi, as well as from frozen skin and melanoma samples, indicate that a differential volatile profile of melanoma does indeed exist.

## Results

### The volatile collection preserves the tissue morphology

**Figure 1:**
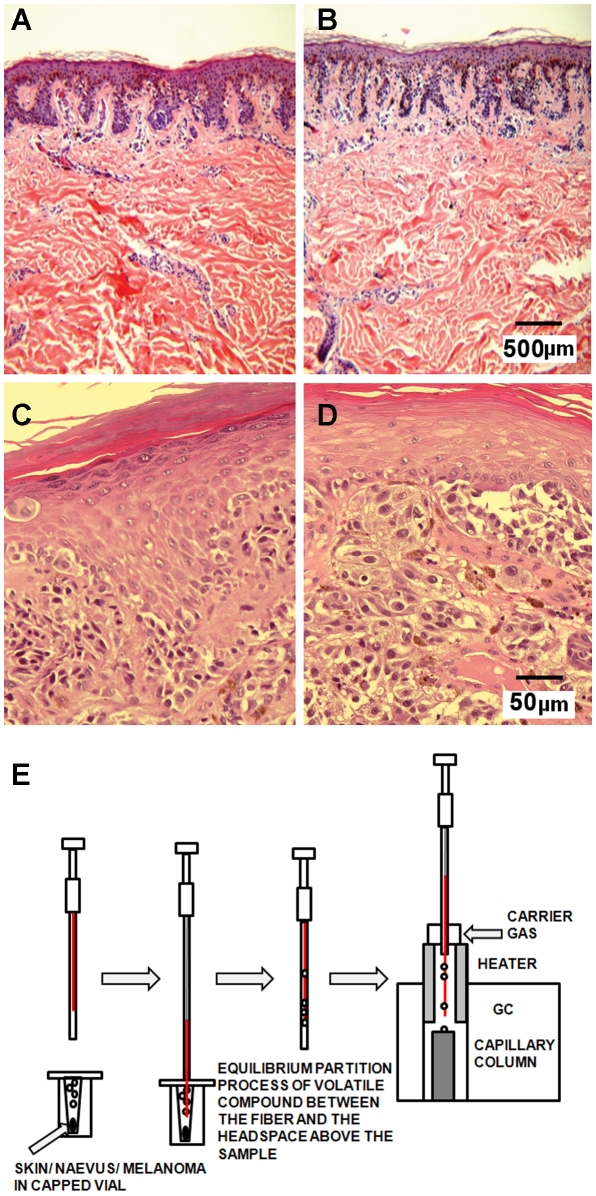
Volatile collection preserves the tissue morphology. H&E staining of naevus (**A** and **B**) and melanoma (**C** and **D**). Histological analysis of the first punch biopsy sample placed immediately in formalin (**A** and **C**). Histological analysis of the second punch biopsy, after collection of volatiles (**B** and **D**). No obvious deterioration of the tissue samples was detected by the histopathologist. **E.** Volatile collection by HS-SPME method. Skin, naevi or melanoma 3-mm punch biopsy sample was placed in a small capped vial. PDMS-DVB fiber (red) was exposed to the head-space above the biopsy sample for 1 hour. After volatile collection, the fiber is retracted, and injected into GC/MS.

The diagnosis of melanoma is based on histological analysis of tissue biopsies and remains the primary modality of detection. Here, we show that our method of volatile collection does not alter tissue morphology. Five naevi samples and three melanoma lesions big enough to obtain *two* parallel 3-mm samples were used for both histology (H&E staining) and volatile collection analysis. The first biopsy sample from each naevus and melanoma was put straight into formalin, embedded in paraffin, sectioned and stained using standard histopathological methods (Figure 1A and C). The second, parallel biopsy sample from each naevus and melanoma was first subjected to the volatile collection by using our HS-SPME method (Figure 1E) and volatile analysis. Less than three hours after biopsy collection and volatile analysis, samples were put into formalin and processed for histology (Figure 1B and D). Because the histological samples from the two groups were indistinguishable, we conclude that our volatile analysis of tissue biopsies does not alter tissue morphology. Thus, our volatile collection does not change tissue appearance and does not interfere with standard clinical procedures related to melanoma diagnosis.

### Comparative analysis of volatile compounds in the fresh naevi and melanoma

**Figure 2:**
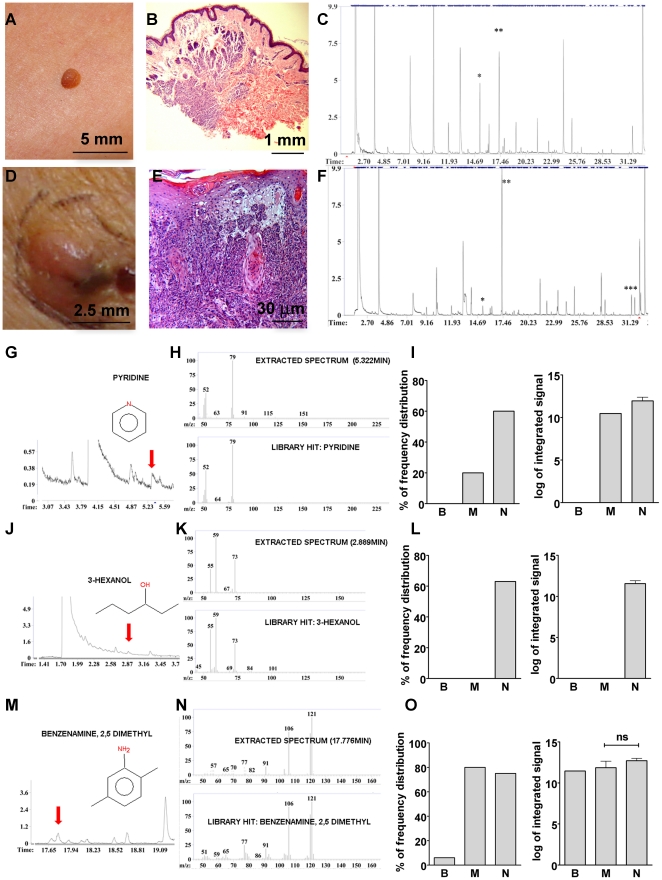
From melanoma and nevus to volatile signatures. Pictures from naevus (**A**) and melanoma skin lesion (**D**), H&E staining of biopsy from naevus (**B**) and melanoma lesion (**E**), chromatograms from naevus (**C**) and melanoma (**F**). Some peaks are unique in melanoma (***), some are increased (**) and some are decreased (*) in melanoma *vs*. naevi. (**G, J** and **M**) Chemical structure of pyridine, 3-hexanol and 2,5 dimethyl benzenamine, and their retention time in the chromatograms (indicated by the arrow). (**H, K** and **N**) Mass spectra of the indicated peaks (extracted spectrum, above) and mass spectra from the library (library hit and identification of the compound, bellow). (**I**, **L** and **O**) Frequency distribution of these three compounds in blank (B), melanoma (M) and naevi (N) group, as well as their expression analysis (log of integrated signal) are presented (t-test, mean±SEM). In (**O**) right panel n = 16 for N and n = 3 for M. Dimethyl benzenamine (2,5; 2,3; 2,4 or 2,6) is a volatile compound present in 19 out of 25 naevi samples, in 4 out of 5 melanoma samples and detected in only one air sample. A peak of 2,5 dimethyl benzenamine is shown eluting at 17.8 min. This compound is common in both melanoma and naevi group.

Demographic data for volunteers and melanoma patients used to obtain fresh tissue, as well as data obtained from the Cooperative Human Tissue Network bank (CHTN) regarding frozen tissue are summarized in the Table S1. In addition, histopathology reports for the 5 fresh melanoma biopsy samples are presented in Table S2. Analysis of the volatile molecules collected from fresh naevi (n = 25) and fresh melanoma samples (n = 5) revealed complex volatile chromatographic signatures (Figure 2, C and F). Some peaks in the chromatograms were only present in melanoma group (indicated with ***), while some were increased (**) or decreased (*) in the melanoma group as compared to the naevi group. In total, using AMDIS (Automated Mass Spectral Deconvolution and Identification software) and the NIST 2.0 mass spectral library, we detected and identified 325 unique volatile compounds from naevi (N) and melanoma (M) samples with ≥60% confidence.

Thirty two compounds were present in 60% of the samples from the naevi group. Most of these compounds were also detected in both melanoma and air samples (blanks). Only pyridine and 3-hexanol appeared to be specific for naevi (Figure 2, G and J). Pyridine is present at 60% frequency in naevi group. It was detected only in one melanoma sample and it was not detected in air samples. 3-hexanol was detected exclusively in the naevi group. We also identified dimethyl benzenamine (at carbons 2,3;2,4;2,5 or 2,6) as a common volatile compound present in both naevi and melanoma (Figure 2, M, N and O). Compounds that differ only in a methyl group position (as is the case with the dimethyl benzenamine) have similar mass spectra and thus are hard to distinguish using gas chromatography/mass spectroscopy.

### Statistical analysis of melanoma differential volatiles identified from fresh biopsy samples

Differential components were statistically identified based on two criteria, the amplitude or the frequency. A difference in amplitude was defined as a statistical significance of the difference in the pair-wise mean value (“Total Integrated Signal” or “area under the peak”) (Student t-test) of expression between groups (naevi “N”, melanoma “M”, and blank “B”, ambient air sample). A difference in frequency was defined as the statistical significance of the difference in the frequency of appearance (Cochran-Mantel Haenszel test). We looked for the relative frequencies of the volatiles in each group and tested their significant difference in distribution by using the odds/ratio (comparing a frequency in M *vs* frequency in N group) and Cochran-Mantel Haenszel test (to determine the likelihood of seeing a compound in the M *vs* N group); an odds ratio of >2.5 and a metabolite present in 40% or more melanoma biopsies indicated a potential biomarker or molecule of interest.

We found 21 volatile compounds present at a significantly different frequency between the two groups (Table 1); among them alkanes (e.g., nonane), methylated alkanes, alkenes (e.g., tridecene) were abundant. Phthalate, butanal, dimethylsulfone and indole, were all 16X more likely to be detected in the melanoma then in the naevi group. We identified the presence of longer chain alcohols, from dodecanol to hexadecanols (C12-C16). Methylated benzenes (compounds 5-10 in Table 1) were also abundant in the melanoma group. Although we listed them in the Table 1, the exact identity, (i.e. the position of the methyl groups) of these compounds is elusive, since their retention times (between 12–13 minutes) and their mass spectra are very similar.

**Table 1:** Volatile compounds with significant differences in frequency distribution in fresh melanoma *vs* fresh naevi samples (Cochran-Mantel Haenszel test, M-melanoma, N-naevi).

No	CAS	COMPOUND	GROUP	M	N	p-value	odds ratio
1	111-84-2	Nonane	aliphatic saturated hydrocarbon	5/5	11/25	0.02	14
2	2847-72-5	Decane, 4-methyl	aliphatic saturated hydrocarbon	3/5	2/25	0.0051	17
3	25377-82-6	1-Tridecene	aliphatic unsaturated hydrocarbon	4/5	7/25	0.03	10
4	5113-87-1	Cyclohexene, 3-methyl-6-(1-methylethenyl)-, (3R-trans)- (E-isolimonene)	unsaturated monocyclic hydrocarbon	4/5	8/25	0.049	9
5	98-82-8	Benzene( 1-methylethyl)	aromatic hydrocarbon	3/5	3/25	0.016	11
6	526-73-8	Benzene, 1,2,3 trimethyl	aromatic hydrocarbon	4/5	7/25	0.03	10
7	95-63-6	Benzene, 1,2,4 trimethyl	aromatic hydrocarbon	4/5	7/25	0.0086	10
8	611-15-4	Benzene, 1ethyl-2-methyl (ethyltoluene)	aromatic hydrocarbon	4/5	5/25	0.049	16
9	620-14-4	Benzene, 1-ethyl-3methyl	aromatic hydrocarbon	4/5	8/25	0.017	9
10	622-96-8	Benzene, 1-ethyl-4-methyl	aromatic hydrocarbon	4/5	6/25	0.017	13
11	100-42-5	Styrene (benzene, ethenyl-)	aromatic hydrocarbon	2/5	1/25	0.016	16
12	85-68-7	Benzyl butyl phthalate	ester	2/5	1/25	0.016	16
13	123-72-8	Butanal	aldehyde	2/5	1/25	0.016	16
14	67-71-0	Dimethyl sulfone	sulfone	3/5	1/25	0.016	16
15	120-72-9	Indole	nitrogen heterocycle	3/5	1/25	0.016	16
16	151-56-4	Aziridine,2-methyl (ethyleneimine)	amine	3/5	3/25	0.01	11
17	112-53-8	1-Dodecanol	alcohol	4/5	8/25	<0.05	9
18	36653-82-4	1-Hexadecanol	alcohol	4/5	8/25	<0.05	9
19	629-76-5	1-Pentadecanol	alcohol	3/5	3/25	0.016	11
20	112-70-9	1-Tridecanol	alcohol	4/5	8/25	0.049	9
21	18172-67-3	B-Pinene	cyclic monoterpene	3/5	3/25	0.016	11

**Figure 3:**
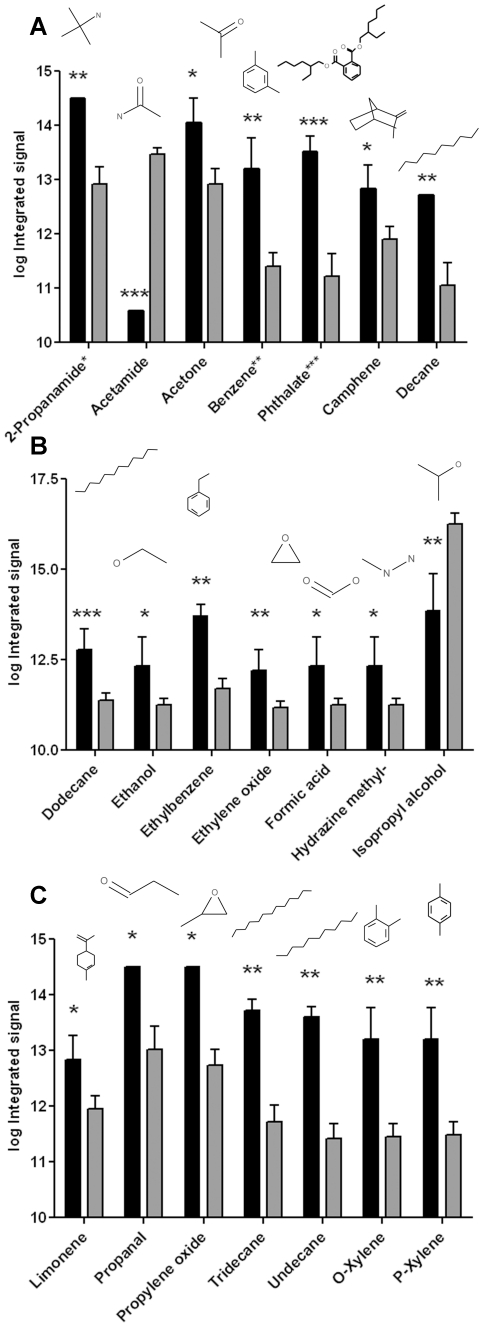
Differentially expressed volatile compounds in melanoma *vs* naevi. The expression level of compounds as indicated by log of integrated signal, in melanoma group (black bars, n = 5) and naevi group (grey bars, n = 25) are shown in panels **A, B** and **C**. Data are expressed as mean ± STDEV, *p = 0.05–0.09, **p = 0.005–0.05, ***p = 0.0001–0.005. In **A**: 2-propanamide* is 2-propanamide, 2-methyl; benzene** is benzene, 1,3 dimethyl and phthalate*** is bis(2-ethylhexyl) phthalate.

The summary of the t-tests, where significant differences in the mean values of the compounds were compared, the associated p-values together with their structures are presented in Figure 3A, B and C. It is interesting to note that only acetamide (Figure 3A) and isopropyl alcohol (Figure 3B) showed decreased levels in the melanoma group relative to the naevi group; all other compounds were found to be significantly increased. Xylene was detected in melanoma but not in naevi. o- and p- xylene have very similar mass spectra and it is difficult to distinguish them by mass spectrometry. Xylene is part of the benzene, toluene, ethylbenzene, o-, m- and p-xylene complex (BTEX), an index of environmental contamination of soil and ground water by petroleum products. Please note that the procedure for collecting volatiles occurs before any histological procedure. Thus, the presence of xylene in melanoma and skin groups (and its absence in naevi) is probably due to intrinsic physiological processes. Indeed, the regular protocol for embedding the tissue in paraffin requires xylene. It is used for clearing the tissue following dehydration step. However, because all of our samples have been analyzed for volatile compounds *before* this step and also because all samples were treated the same way, the presence of xylene in the melanoma and skin groups is *not* due to technical or methodological artifacts.

In summary, the increased presence of methylated alkanes and benzenes may indicate an increased methylation process in melanoma. The presence of secondary metabolites of membrane lipid peroxidation, e.g alkanes (nonane, decane, undecane, dodecane, tridecane), alkenes (decene, tridecene), aldehydes (propanal, butanal) may be an indicator of oxidative stress [7], [8].

### Statistical analysis of differential volatiles from frozen melanoma and skin samples

The availability of the fresh melanoma tissue is often a limiting step in the experimental work. Thus, other sources, such as frozen tissue, need to be considered and explored. With this in mind, 18 frozen melanoma and 20 frozen skin samples obtained from the CHTN tissue bank were collected and analyzed. Similar to our work with fresh samples, analysis of frozen melanoma and frozen skin revealed differential volatile profiles in the two biopsy sets. Demographic data and histopathology reports for frozen melanoma tissue samples were summarized in the Table S3. In total, 220 compounds in the two sample sets were detected; this is less than the 325 detected in fresh samples. Out of 18 melanoma samples, only 3 were skin biopsy samples, the remaining samples were metastatic melanoma tissue from: lymph node (10), intestine (2), liver (1), lung (1) and breast (1). Compounds listed in Table 2 were present at a significantly increased frequency distribution in the melanoma group *vs* the skin group. Compounds listed in Table S4 are volatiles detected at a significantly higher distribution frequency in skin samples. Xylene was detected in the frozen skin samples. We also detected this compound in fresh melanoma, but not in the fresh naevi group (see Discussion). Volatiles with significantly increased mean value and their associated p-values in melanoma *vs* skin group are presented in Table 3. Two compounds, dodecane and 5-methyl dodecane, were significantly increased in melanoma using either the expression level (Table 3) or the frequency of distribution (Table 2).

**Table 2:** Six volatile compounds with significant increase in frequency distribution from frozen melanoma (M-melanoma, S-skin).

No	CAS	COMPOUND	M	S	p-value
**1**	2847-72-5	Decane,4-methyl	18/21	4/20	<0.0001
**2**	112-40-3	Dodecane	21/21	11/20	0.0006
**3**	17453-93-9	Dodecane, 5-methyl	19/21	11/20	0.0114
**4**	142-91-6	Isopropyl Palmitate	14/21	2/20	0.0083
**5**	629-62-9	Pentadecane	9/21	1/20	0.005
**6**	1120-21-4	Undecane	18/21	3/21	<0.0001

**Table 3:** Volatile compounds with significantly increased mean value (log integrated signal) in the frozen melanoma group (t-test).

No	CAS	COMPOUND	P-VALUE
**1**	104-76-7	1-Hexanol,2-ethyl	0.0125
**2**	107-18-6	2-Propen-1-ol	0.0002
**3**	5400-75-9	2H-Benzimidazol-2-one, 1,3-dihydro-5-methyl-	<0.0001
**4**	20633-03-8	3,4-Hexanedione, 2,2,5-trimethyl-	<0.0001
**5**	117-81-7	Bis(2-ethylhexyl) phthalate	0.0087
**6**	138-86-3	D-Limonene	0.052
**7**	112-40-3	Dodecane	0.003
**8**	17453-93-9	Dodecane,5-methyl	<0.0001
**9**	222866	Oxime-,methoxy-phenyl	0.0006
**10**	2078-54-8	Propofol	<0.0001
**11**	4254-14-2	Propylene Glycol	0.0087
**12**	32487-71-1	Pyrrole-3-carbonitrile, 5-formyl-2,4-dimethyl-	0.0021
**13**	90-43-7	o-Hydroxybiphenyl	0.014

### Fuzzy logic-based statistical analysis of the frozen tissue bank samples

**Figure 4:**
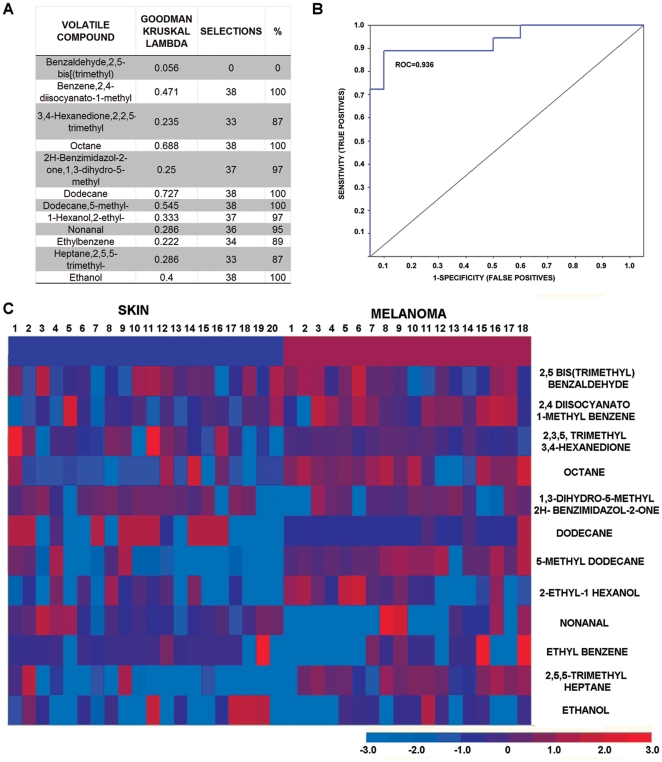
Fuzzy logic analysis of frozen skin and melanoma samples. **A**. list of the volatile compounds, their Goodman Kruskal Lambda values, the number of selections in all (38) leave-one-out runs, and the percentage of how often they were selected. **B**. Receiver operating characteristic curve (ROC). **C**. Heat map for the frozen data. Each column represents one sample. Each row represents one compound. Red colors represent retention time (RT) values that are high above the average; blue colors represent RT-values that are low and much below average. The first row represents the category; whether the sample belongs to the skin samples (left 20 columns with blue color in the first row) or to the melanoma samples (rights 18 columns with red color in the first row). The light blue color represents a missing value. Misclassified in the leave one out method are samples 4 and 12 from skin group, and samples 14 and 18 from the melanoma group.

Retention times from the chromatograms obtained from frozen skin and melanoma samples were used to create a table of test samples from which volatile compounds relevant for the discrimination between the skin and melanoma groups were derived. From a total of 38 samples (18 melanoma and 20 skin samples), twelve volatile compounds were identified as relevant and a fuzzy logic prediction algorithm was created. The list of these compounds with their relevant Goodman Kruskal Lambda value is presented in Figure 4A. A higher Goodman Kruskal Lambda value indicates a higher likelihood that a volatile compound is predictive for melanoma. These potential candidate markers were evaluated by fuzzy logic analysis and demonstrated the ability to distinguish melanoma from skin with 89% sensitivity (16/18) and 90% specificity (18/20). Nonanal is a common and abundant compound found on human skin (Table S4, p<0.0001, see Discussion). Fuzzy logic analysis identified this volatile as one of the 12 compounds that can be used to distinguish melanoma from skin (Figure 4). Leave-one-out cross validation with variable selection in each leave-one-out run was used to assess the predictive performance of the fuzzy logic analysis. The area under the Receiver-Operating characteristic Curve (ROC) is determined as 0.936; indicating that these 12 compounds are able to correctly classify melanoma with high sensitivity and specificity (Figure 4B). A graphical representation of these data is presented in the form of the heat map (Figure 4C).

### Comparison of fresh and frozen volatile signatures in melanoma biopsies

**Figure 5:**
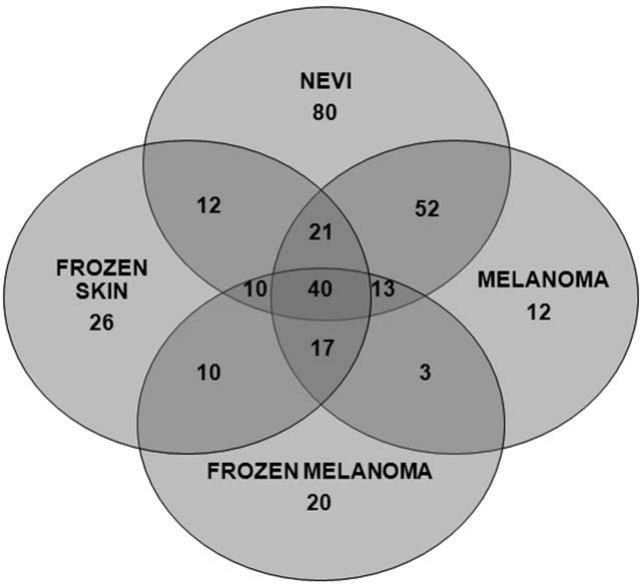
A Venn diagram showing the number of volatile compounds specific for each tested group as well as the numbers of overlapping volatiles between the groups (e.g., naevi has 80 volatiles not expressed in any other group).

In Figure 5 the number of volatile compounds detected in each group, as well as the number of compounds that overlap between fresh naevi, fresh melanoma, frozen skin and frozen melanoma are presented as a Venn diagram to illustrate the complex relationship between the volatile fingerprints of the different sample sets. We detected a total of 35 compounds unique to melanoma, and 3 compounds unique to and common to both fresh and frozen melanoma samples. The availability of the fresh melanoma tissue for research is limited and we sought to investigate whether the frozen tissue will be suitable for this type of study. We documented a differential expression of volatiles in frozen and fresh melanoma samples as compared to the controls; however we did not observe the complete overlap in these two sets of melanoma volatile profiles. This was probably due to a difference in the type of control tissue used and in the tissue preparation (fresh or frozen). Although volatile expression profiling from frozen tissue has a limited potential for clinical utility, here it served to demonstrate the proof of principle.

In case of 3 volatile compounds tissue preparation methodology (freezing/thawing) did not have an effect. Thus, despite the differences in methodology, three compounds identified in fresh tissue were still predictive of melanoma in frozen tissue. 4-methyl decane, dodecane and undecane were detected from both fresh and frozen melanoma at a significant level compared to the control group; thus these compounds are potential candidate biomarkers. 4-methyl decane was present at significantly increased frequency, while dodecane and undecane were present at significantly increased both frequency and expression level. Dodecane was also one of the 12 candidate volatiles identified by fuzzy logic analysis. However, methylated benzenes, dimethyl sulfone, ethyleneimine, nonane, indole and longer chain alcohols initially detected in fresh melanoma samples, together with some aldehydes (propanal, butanal) were not found in our frozen samples, indicating that the volatile signature may have changed due to the freezing process and prolonged storage. The results from our frozen melanoma group have a different control, skin instead of naevus (used as a control in our fresh group), precluding a full comparison; however if a volatile is to be a true melanoma biomarker, its presence should be detected neither in skin, nor in nevus.

## Discussion

In this paper we presented a novel approach for detecting metabolites of melanoma and demonstrated a proof of principle that a differential metabolic signature of melanoma does indeed exist. Our results support the hypothesis that volatile metabolites change as a result of cancerous process. This altered volatile signature has the potential to develop into a new diagnostic tool. It is particularly important for melanoma, since early detection of melanoma is critical for a positive outcome for patients. Our results indicate that combining HS-SPME with GC/MS to detect volatile signatures from naevi and melanoma tissue is a valid approach. A similar approach has been used to detect volatile organic compounds (VOC) in breath of lung [9], [10], [11], [12] and breast cancer [13] and melanoma patients [14]. In this study, we showed that after volatile collection tissue can be used for histology analysis.

The current prevailing opinion is that many melanomas arise *de novo* and only a subset of melanoma arises from the naevus [15], [16]. In the current study, we used acquired melanocytic naevi (i.e. moles) from volunteers as a control group to which our melanoma samples were compared. Naevi are benign tumors of melanocytes with an oncogene-induced senescent phenotype. Naevi exist in a growth-arrested state predominantly induced by BRAF^V600E^ [17]. There is no literature record of volatiles released from naevi, and this study is the first to address this question. Although most volatiles present in naevi were also present in the melanoma group, the level of these volatiles detected was significantly different for 21 compounds (Figure 3). In addition, the volatile signature from naevi revealed pyridine and 3-hexanol as specific naevi compounds not found in melanoma (Figures 3 and 5).

The presence of pyridine implies a unique metabolism in naevi. The number of naevi present during childhood is a predictor for melanoma and it can also be an indicator of an early harmful UV exposure, or both [18]. UV exposure, oxidative stress and reactive oxygen species (ROS) induce breaks in DNA [19]. PARP-1 [Poly(ADP-ribose) polymerase-1] an enzyme involved in DNA repair stimulates polymerization of ADP-ribose [20]. ADP-ribose polymers are rapidly turned over and converted to free ADP-ribose by the action of poly (ADP-ribose) glycohydrolase (PARG) [21]. In this reaction NAD (nicotinamide adenine dinucleotide) is used as a substrate and nicotinamide (a pyridine derivative) and free ADP-ribose are produced. Thus, prolonged oxidative stress depletes the NAD content of the cell [22]. Any metabolic event which results in the breakdown of an NAD molecule requires re-synthesis of NAD to maintain intracellular levels of this co-enzyme. It is called Pyridine Nucleotide Cycle [23]. We postulate that in the naevi, similar processes occur that may explain the metabolic origin of pyridine as a nicotinamide derivative and a unique volatile biomarker of naevi.

Analysis of volatiles from fresh melanoma tissue clearly indicates a differential metabolic signature. The metabolic origin of most volatiles detected in this study is complex and remains elusive. Some of these compounds have also been reported as volatile organic compounds (VOC) associated with cancer. Acetone (Figure 3A) and styrene (Table 1) were significantly increased in our fresh melanoma group and these two volatiles have also been found in the blood of lung cancer patients [7]. Undecane was detected in both fresh and frozen melanoma samples (Figure 3C and Table 3), and has been reported as a potential marker for lung cancer [24].

Our statistical analysis indicates that nonane (detected in all five fresh melanoma samples) is 14 times more likely to be present in melanoma, and this volatile was also identified as one of 8 breast cancer biomarkers [13].

Increased levels of indole indicates increased melanin synthesis [25]. Consistent with this, a recent study describing melanin composition analysis (pyrolysis in combination with GC/MS) reported styrene, benzene, pyrole, phenol, indole and their alkyl derivatives as main compounds formed during this thermal degradation [26].

Literature data on volatiles released from skin does exist [27], [28]. A comprehensive study of human skin volatile compounds identified mostly carboxylic acids. In addition C3-C9 aldehydes; alkanes and alkenes; C4-C10 ketones and many heterocyclic structures detected [27]. Skin, the largest body organ, is exposed to many environmental toxins that are either oxidants themselves or produce reactive oxygen species (ROS) [29]. Aldehydes in the skin are generated by the oxidation of unsaturated fatty acids released from membrane phospholipids by UV generated ROS. Consistent with these previous studies, in the current study using frozen tissue bank samples, we detected many aldehydes from frozen skin and in particular nonanal (see Table S4). Nonanal is formed by the oxidative degradation of sebaceous component, mainly oleic acid [30].

Xylene has been previously identified as skin volatile compound [27]. It is part of the BTEX compounds, that are gaining more attention as these compounds are found to be ubiquitous pollutants both indoors and outdoors [31]. D-limonene is significantly increased in both fresh and frozen melanoma, however, it is likely that D-limonene is an air and/or cosmetic contaminant because this compound was detected in blank air samples (Table S5), and also in the frozen skin samples (Table S4).

Fuzzy logic analysis is a powerful methodology. Analysis of frozen skin and melanoma revealed 12 potential biomarkers with 89% sensitivity and 90% specificity. A concern here is that the biopsy collection procedure is unknown (samples were obtained from CHTN, for details see Material and Methods section, Tissue Collection) and may contribute to the unique volatile signature. Any systematic difference in the collection of the two types of samples could cause a different volatile profile. In addition, prolonged storage of these frozen samples (up to one year) may result in differential profile. However, one compound deserves special attention, dodecane. This simple alkane was detected in both fresh and frozen melanoma by all statistical tests used (Chi-square test, t-test and fuzzy logic). This compound is a potential candidate marker of melanoma in addition to 4-methyl decane, and undecane. Further studies on large number of patients will be necessary to confirm it. These alkanes may be products of increased oxidative stress and lipid membrane peroxidation.

The methylated alkane, 4- methyl decane, was listed as one of 16 biomarkers of lung cancer [10]. The detection of methylated volatiles from melanoma suggests increased methionine metabolism [32]. Increased levels of dimethyl sulfone (Table 1) and formic acid (methanoic acid, Figure 3B) were detected with HS-SPME from fresh melanoma samples. Previous studies have demonstrated a higher expression of methionine cycle genes in melanoma [33]. Methionine is the major source of the methyl groups necessary for the methylation of DNA and other molecules [34] and “methionine dependence” (absolute requirement of methionine) is a phenotype characteristic of tumor cells [35]. S-adenosyl methionine (SAM) carries and donates methyl groups to facilitate cellular replication and detoxification reactions. The human metabolome database (HMDB) lists 118 methyltransferases, enzymes that can use SAM as a methyl donor. Certain classes of the large and diverse family of methyltransferases (I-III) have very diverse substrates that include amines, alcohols, carboxylic acids, thiols, sulfides, alkenes and alkanes [36], [37]. Aberrant DNA methylation represents a common mechanism of transcriptional silencing seen in cancer and is also present in melanoma [38]. It has been shown that cancer cells are subject to abnormal *de novo* methylation [39]. Increased levels of DNA methyltransferases were detected in some cancers [40], [41]. By using a bioinformatics tool, Oncomine Concept Map (OCM, http://www.oncomine.org) Sreekumar et al. [42] predicted increased “methyltransferase activity” among the metabolites up regulated in metastatic prostate cancer. The highly methylated environment in human melanoma cell lines (e.g., SkMel-28) has been reported, and both COMT (Cathechol O-methyl transferase) and DNA methyltransferase-1 were found to be increased approximately five and eight times, respectively [33]. Furthermore, it was published previously that COMT activity is present in the skin [43], where it is involved in the methylation of the o-dihydroxy phenolic and o-dihidroxyindolic melanogens, thus reducing their redox cycling and the generation of free radicals [44]. A few methylated amino-acids have been recently revealed in connection with cancer. Increased levels of methyl glycine or sarcosine in plasma of prostate cancer patients has been shown to be associated with its progression to metastasis [42]. Also, methyl alanine has been identified as one of the candidate biomarkers for pancreatic ductal adenocarcinoma [45]. Our results show an increase in methylated aromatic hydrocarbons (benzenes) and alkanes in melanoma. This is the first evidence of methylation of small molecules or metabolites that has been reported in connection with melanoma. With the advent of metabolomics technologies and the increased interest in metabolomics, we predict that more methylated metabolites will be found, especially in the context of melanoma and cancer. Comprehensive volatile metabolomic studies might also help in improving melanoma classification. Finding a correlation between volatile molecular signatures and clinical parameters of melanoma will complement recent genotype-phenotype studies [46], [47] and ultimately lead to an improved targeted therapy.

## Materials and Methods

### Tissue collection

We obtained ethics approval from the Institutional Review Board at the University of Miami and the Miami VA Healthcare system. Biopsy samples were obtained from subjects recruited in accordance with an approved University of Miami Institutional Review Board (IRB) protocol (No. 2006117) and Veteran Administration IRB protocol (No. 00762). A written consent form was obtained from each subject. All naevi samples were collected from the volunteers (asked not to wash 8 hours before the biopsy) and were confirmed by histology analysis (by G. Elgart) using hematoxylin & eosin staining. Each naevus was removed by using a 3-mm punch device (AcuPunch, Acuderm, Inc). Fresh melanoma samples were collected from patients scheduled for the excisional biopsy irrespective of histotype or disease stage. No exclusion criteria were used, except that all samples were from patients over 21 years of age. The melanoma lesion was first excised and then cut with a 3-mm punch device in order to obtain the same sample size as for naevi. The reason why each melanoma lesion was first excised and then cut using the punch biopsy technique is because excisional biopsy of melanoma is a preferred method to remove a malignant lesion [48]. We did not want to compromise the safety of patient for research purposes. Immediately after the lesion was removed, a 3-mm punch biopsy on the excised tissue was performed for our research purposes. We do not believe that punch biopsy of melanoma tissue immediately after excision of the tissue introduces any artifact that could account for the sampling difference. After biopsy and volatile collection, melanoma samples were analyzed and confirmed by H&E staining. We also collected and analyzed 17 control air samples to identify air contaminants. The air was collected from the same room where biopsy of naevi samples took place and the whole procedure was identical to the one with biopsy tissue. In addition, we obtained chromatograms from the anesthetic used in the biopsies (1% Xylocaine/Epinephrine & Na-bicarbonate). Eighteen frozen melanoma and twenty frozen skin samples were obtained from the CHTN tissue bank (Cooperative Human Tissue Network). Prior to storage at the tissue bank these samples were snap frozen (in liquid N_2_) within 1 hour after biopsy to quench metabolism, and kept at −70°C for not more than one year. Frozen samples were also cut with the 3-mm punch device to obtain a uniform size, placed in the 1.5 mL vial and thawed on ice for 1 hour [49] and later processed in the same way as fresh naevi and melanoma samples.

### Histology analysis

Hematoxylin/eosin staining was done at the Dermatopathology Laboratory Services Department of Dermatology University of Miami, Miller School of Medicine. All fresh and frozen samples after volatile collection were put into formalin and confirmed by histology analysis done at the Department of Pathology, Jackson Memorial Hospital.

### HS-SPME collection of volatiles

We used HS-SPME (head space-solid phase microextraction) method to collect the volatiles [6], [50], [51]. This method uses a small, portable device with a coated fiber to extract and collect volatile compounds for analysis by gas chromatography. The biopsy sample was placed in a vial (Agilent, No. 5182-0715, 1.5 mL, with 0.3 mL polyspring insert) and capped with a Teflon coated silicone septum (Figure 1E). The sample was kept refrigerated for not more than one hour. After that the sample was left at room temperature for one hour to equilibrate. The headspace was sampled with a polydimethysiloxane-divinylbenzene fiber for one hour at room temperature (65-µm PDMS-DVB, Cat No. 57344-U, Supelco, Bellefonte, PA, USA). Extraction selectivity depends on the type of the fiber and the coating thickness. Since we are dealing with an unknown complex matrix, we decided to use a fiber with broad selectivity, non-polar PDMS/DVB. In order to achieve higher sensitivity, the sample headspace should be as small as possible [6], therefore we introduced the inner tube into the 1.5 mL vial. We also decided to use a fiber of 65 µm, of medium thickness; a general rule is that a thick fiber adsorbs more compounds; however their diffusion from the fiber during thermal desorption is slower. Analysis of air samples obtained in parallel with biopsy collection (n = 17) revealed 34 compounds that were present in at least 40% of the samples (Table S5). Some components known to elute from the fiber itself were excluded from this list (largely siloxanes and their derivatives). Volatile analysis of the anesthetic lidocaine used in the biopsy procedure of nevi samples revealed the presence of additional volatiles: oxime-, metoxy-phenyl and methylparaben. Lidocaine (5 µL) was placed in the vial, and the volatiles were collected in the same fashion as described for tissue samples in the Material and Methods section. In one biopsy of melanoma, in addition to lidocaine, midazolam, propofol and fentanyl were used as anesthetics. We analyzed volatiles from these anesthetics and detected benzene, 2,4 diisocyanato-1-methyl and diphenyl ether in addition to original anesthetics and the above mentioned volatiles from lidocaine. No information was available about anesthetics used to obtain frozen samples from CHTN. Compounds like propofol and methoxy-phenyl oxime (Table 3), as well as 2,4 diisocyanato-1-methyl benzene (Figure 4A) were most likely released from anesthetics used in biopsy procedure.

### Optimization of the HS-SPME conditions

The parameters such ***fiber coating*** (75 µm PDMS/Carboxen Cat No. 57284-U and 65 µm PDMS/DVB), ***sample size*** (2- and 3-mm) as well as ***post biopsy time of volatile collection*** were investigated (Figure S1). PDMS/Carboxen is predominantly used for relatively small volatiles, compounds between 2–10 carbons and low MW compounds (MW = 30–225), while PDMS/DVB is used for volatiles, amines, nitro-aromatic compounds, ranging from 50–300 Daltons. We analyzed two 3 mm biopsy samples from the same nevus with both fibers (TIC, total ion count, for PDMS/DVB is about 91535, while for the PDMS/Carboxen is 70127) (Figure S1A). By comparing these two chromatograms we observed that the difference is mainly quantitative and not qualitative. The AMDIS postprocess comparison of these two files is presented in the Figure S1B. From the 3-mm sample we detected about 20 times more signal (Total Ion Count, TIC) than from the 2-mm sample. Also, the number of identified compounds was much larger (344 *vs* 260) (Figure S1C). Thus, in all subsequent experiments, we performed a 3-mm punch biopsy. To examine the effect of post biopsy collection time on volatile compounds, *the axilla sample from melanoma patient* was collected and punched twice; the first sample was analyzed within 3 hours of biopsy, while the second sample was kept refrigerated overnight and analyzed the next day. The integrated signal for each compound was divided by the sum of signals from the whole sample (TIC) and the percent of each volatile compound was calculated (Figure S1D). Comparison of these two samples indicates loss of volatile compounds of about 30% in TIC. In the first sample, we identified 21 compounds, while in the second we identified 25 compounds. Out of 17 compounds that were detected in both samples, eight showed increase of ≥2 fold after 24 hours. These compounds were: 1-Hexanol 2-ethyl-; 3-Methylbenzothiophene; Acetic acid butyl ester; Diethyl Phthalate; Hexane 3,3-dimethyl-; Limonene; Oxime- methoxy-phenyl-l and Phenol 2,4-bis(1-methylethyl)- acetate. Volatile compounds that couldn’t be detected in the 24 hours/second sample, and thus were lost were: Benzene 1,4-dichloro-, Dodecane, Ethanol and Methyl-hydrazine. The difference in volatile profile between these axilla samples indicate that degradation process is indeed happening, even if the sample was kept refrigerated overnight. In addition we analyzed the volatile composition of two fresh skin melanoma samples within three hours and after 6–8 hours *post biopsy* (data not shown). Approximately 15% of compounds could not be detected after the prolonged incubation at room temperature. Disappearance of some volatiles after prolonged incubation indicates a need for a stringent control of experimental conditions, in particular volatile collection time *post* biopsy. Thus, in all fresh tissue samples (melanoma and naevi) reported in this paper volatiles were collected and analyzed within three hours *post* biopsy.

### Gas Chromatography/Mass Spectrometry (GC/MS)

The PDMS/DVB fiber with collected volatiles was directly injected onto a 0.75 mm i.d. injection port of Hewlett Packard 6890 gas chromatograph (Hewlett Packard, Avondale, PA) and chromatographed on a non-polar DB-5MS column (model No. J&W 128–5522, 25 m ×0.2 mm i.d.x0.33 µm film) under the following temperature program: 40°C for two minutes followed by 6°C min^−1^ ramp to 270°C and hold for 5 minutes. Helium carrier gas flow was run in constant flow at 0.7 mL min^−1^. As volatile compounds elute from the column, they were fragmented into ions (by electron ionization) and detected in the quadrupole mass spectrometer. Each compound produced a unique spectrum of molecular fragments (ions) with specific masses and a fixed relative abundance. We used the Agilent 5973 mass spectrometer in the full scan mode (30–300 amu).

### Preprocessing of the data

GC/MS metabolic profiling results in complex chromatograms with huge differences in the relative abundance of different compounds and with many co-eluting peaks that have to be deconvoluted. We used the AMDIS deconvolution algorithm freely available at www.amdis.net (Automated Mass Spectral Deconvolution and Identification software). Deconvolution finds ions whose individual abundances rise and fall together over time, indicating that they are from the same compound. AMDIS parameters were: 60% minimum matching factor, threshold-low, resolution-medium, sensitivity-high, shape requirements-medium, adjacent peak subtraction-two, low m/z 50, high m/z 300. AMDIS generates a report file, where area under each peak represents the total absolute amount of ions from each compound/metabolite present. Our profiling quantifies metabolites based on their absolute mass ion intensity. This eliminates the need for internal standards and makes measurements of both known and novel metabolites possible [52], [53]. All components from AMDIS analysis were searched in the NIST database (with one reported hit per compound and with a minimum match factor set to 60%, meaning that a threshold of 60% similarity was used for the spectral matching. After generating an analysis report in the text file, we transferred the annotated compound list to EXCEL for further processing. For each sample identity of the compound; CAS or NIST number; retention time and integrated signal were reported. Signal intensity represents the log10 transformation of absolute ion counts in the area under the deconvoluted peak (Integrated signal), thus we were able to compare signals from multiple samples.

### Data analysis

We used the Student t-test and Cochran-Mantel Haenszel Chi-square test to detect statistical significance between our studied groups. In addition, for analysis of frozen melanoma and skin samples we used fuzzy logic methodology by Interrelation Miner software from SystAim (http://www.interrelationminer.com). The Interrelation Miner methodology analyses the relations between the measured variables/volatiles statistically and constructs fuzzy functions for every of these interrelations. With these fuzzy functions, the software creates the membership matrix for each group of samples such as the membership matrix for the group of samples with Disease Present and membership matrix for the group of samples with the Disease Not Present. Using the membership matrix of each group of samples it can be calculated how typical a sample is for each group. The prediction is simply the group with the highest fuzzy membership.

## Supporting Information

Figure S1 Optimization of the HS-SPME conditions. (A) Effect of different fiber coatings (PDMS/Carboxen and PDMS/DVB) on total ion count (TIC). (B) Comparative analysis of two different chromatograms obtained with different fiber coatings from A. (C) Effect of sample size on total ion count (S/N ratio ≥5) (D) Change in % of TIC for volatile compounds analyzed from the same axilla sample (two biopsies) within 3 hours (black) and after 24 hours of biopsy (red) (sample was kept at +4°C).
(2.26 MB TIF)

Table S1 Demographic data for volunteers and melanoma patients used in this study (W-White, B-Black, IND-Indian).
(0.01 MB DOCX)

Table S2 Demographic data and histopathology reports for melanoma patients used to obtain fresh biopsy samples.
(0.01 MB DOCX)

Table S3 Demographic data and histopathology reports for frozen melanoma tissue samples.
(0.01 MB DOCX)

Table S4 Volatile compounds with significant increase in frequency distribution from frozen skin (M) melanoma, S-skin. (*-N/A).
(0.01 MB DOCX)

Table S5 Common compounds present at ≥40% of control air blank samples.
(0.01 MB DOCX)
