# Supplementary figures and images for: Differential Volatile Signatures from Skin, Naevi and Melanoma: A Novel Approach to Detect a Pathological Process
Source: PLoS One. 2010 Nov 4;5(11):e13813. doi: 10.1371/journal.pone.0013813 (PMC2973952; doi:10.1371/journal.pone.0013813)

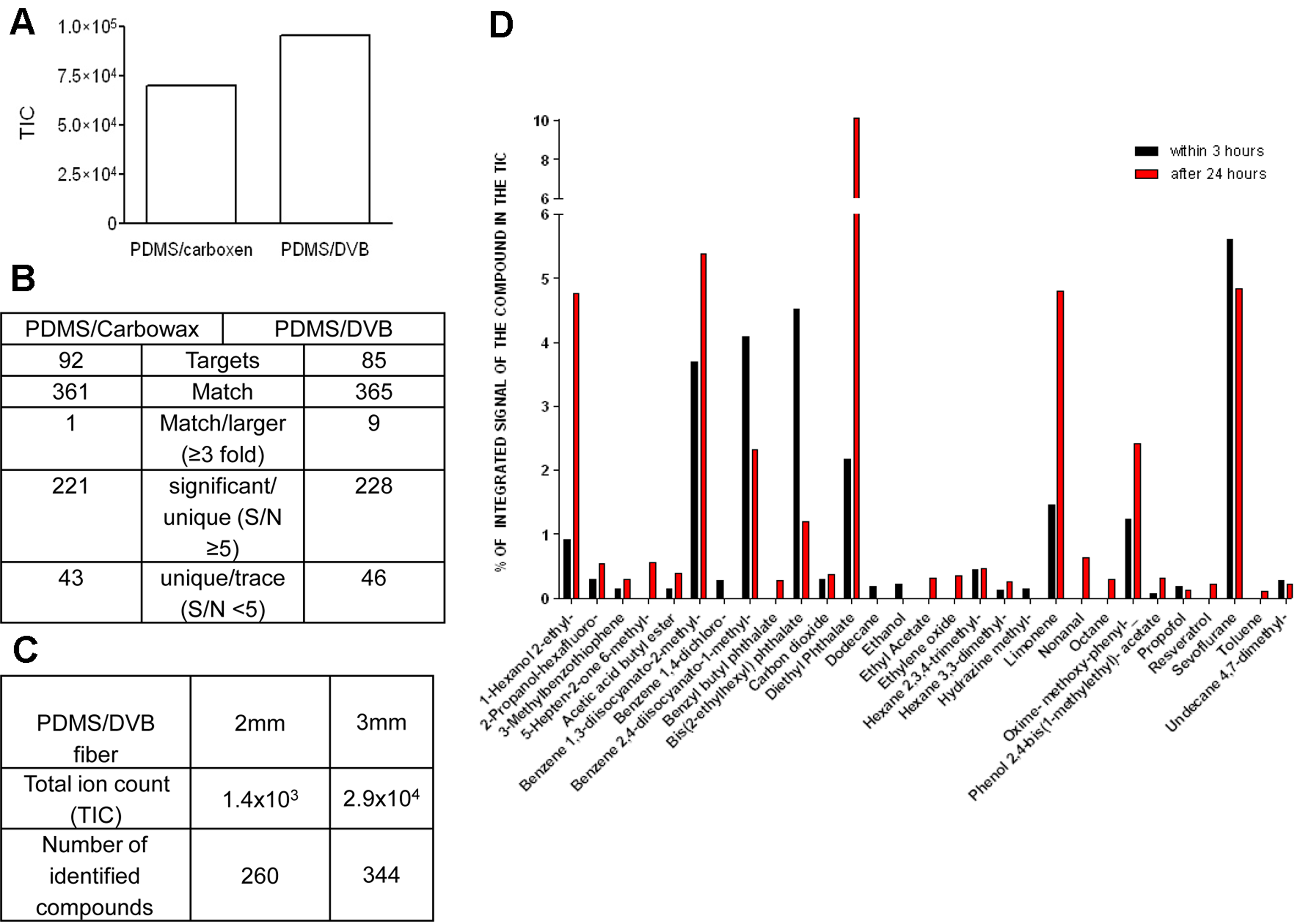

Supplement: Figure S1 — Optimization of the HS-SPME conditions. (A) Effect of different fiber coatings (PDMS/Carboxen and PDMS/DVB) on total ion count (TIC). (B) Comparative analysis of two different chromatograms obtained with different fiber coatings from A. (C) Effect of sample size on total ion count (S/N ratio ≥5) (D) Change in % of TIC for volatile compounds analyzed from the same axilla sample (two biopsies) within 3 hours (black) and after 24 hours of biopsy (red) (sample was kept at +4°C). (2.26 MB TIF) [file pone.0013813.s001.tif]
