# Supplementary material for: Differential Volatile Signatures from Skin, Naevi and Melanoma: A Novel Approach to Detect a Pathological Process
Source: PLoS One. 2010 Nov 4;5(11):e13813. doi: 10.1371/journal.pone.0013813 (PMC2973952; doi:10.1371/journal.pone.0013813)
Supplement: Table S1 — Demographic data for volunteers and melanoma patients used in this study (W-White, B-Black, IND-Indian). (0.01 MB DOCX) [file pone.0013813.s002.docx]

| **GROUP** | **No** | **AGE** | **SEX** | **RACE** | |
| --- | --- | --- | --- | --- | --- |
| **nevi** | 25 | median 40 (21-64) | 10F 17M | 24 W 1 IND |  |
| **fresh melanoma** | 5 | median 68 (61-98) | 3F 2M | 5W |  |
| **frozen melanoma** | 18 | median 55 (28-79) | 7 F 11M | 17W 1B |  |
| **frozen skin** | 20 | median 46 (28-73) | 18F 2M | 16W 4B |  |
