# Supplementary material for: Differential Volatile Signatures from Skin, Naevi and Melanoma: A Novel Approach to Detect a Pathological Process
Source: PLoS One. 2010 Nov 4;5(11):e13813. doi: 10.1371/journal.pone.0013813 (PMC2973952; doi:10.1371/journal.pone.0013813)
Supplement: Table S2 — Demographic data and histopathology reports for melanoma patients used to obtain fresh biopsy samples. (0.01 MB DOCX) [file pone.0013813.s003.docx]

| **No** | **AGE** | **SEX** | **HISTOPATHOLOGIC TYPE** | **LOCATION** | **BIOPSY SAMPLE** |
| --- | --- | --- | --- | --- | --- |
| **1** | 88 | F | Malignant melanoma - Superficial spreading melanoma | right arm | Skin metastasis |
| **2** | 98 | F | MM, Clark level IV, Breslow thickness 1.03cm, Nodular melanoma | left arm | Skin  metastasis |
| **3** | 61 | M | Malignant melanoma (S-100 and HMB-45 positive) | ear | Primary melanoma |
| **4** | 68 | F | Recurrent Malignant melanoma in subcutaneous nodules | right arm | Skin Metastasis |
| **5** | 64 | M | Malignant Melanoma in situ | right chest | Primary melanoma |
