# Supplementary material for: Differential Volatile Signatures from Skin, Naevi and Melanoma: A Novel Approach to Detect a Pathological Process
Source: PLoS One. 2010 Nov 4;5(11):e13813. doi: 10.1371/journal.pone.0013813 (PMC2973952; doi:10.1371/journal.pone.0013813)
Supplement: Table S3 — Demographic data and histopathology reports for frozen melanoma tissue samples. (0.01 MB DOCX) [file pone.0013813.s004.docx]

| NO | DIAGNOSIS | TISSUE ANALYSED | COMMENTS | Sex | Age | Race |
| --- | --- | --- | --- | --- | --- | --- |
| 1 | metastatic MM (Clark's level V) | soft tissue, right axilla (lymph node) | spindled, mostly amelanotic cells | M | 63 | W |
| 2 | metastatic MM (Clark's level IV ) | lymph node, right cervical | S-100 and MART-1 positive | F | 65 | W |
| 3 | metastatic MM | lymph node, left neck |  | M | 56 | W |
| 4 | metastatic MM | lung, left lower lobe | S-100, MART-1 and HMB45 positive | M | 55 | W |
| 5 | metastatic MM | lymph node |  | M | 75 | W |
| 6 | metastatic MM | small intestine |  | M | 46 | W |
| 7 | metastatic MM | skin | extensive necrosis | F | 57 | W |
| 8 | MM, Breslow thickness 4mm, Clark level V, ulceration present | skin from left arm | Melan-A, S100 and HMB45 positive; SMA and desmin negative; CD68 positive | M | 79 | W |
| 9 | MM, ulceration present | lymph node, left neck | MM involving adipose tissue | M | 53 | W |
| 10 | MM, nodular type, level V, Breslow thickness 4.5mm, ulceration present | skin, left shoulder | satellite nodules with lympho-vascular and perineural invasion, mitotic activity 15/mm2 | F | 40 | B |
| 11 | metastatic MM, Beslow thickness 5mm | liver | spindle cell neoplasm | M | 49 | W |
| 12 | metastatic MM | lymph node, left axillary |  | M | 63 | W |
| 13 | metastatic MM | lymph node |  | F | 83 | W |
| 14 | metastatic MM | lymph node, left superficial inguinal |  | M | 76 | W |
| 15 | metastatic MM | lymph node | Melan-A and S-100 positive, HMB-45 negative | F | 40 | W |
| 16 | metastatic MM | breast |  | F | 47 | W |
| 17 | metastatic MM | soft tissue, left axilla |  | M | 28 | W |
| 18 | metastatic MM | metastasis to colon |  | F | 54 | W |
