# Supplementary material for: Differential Volatile Signatures from Skin, Naevi and Melanoma: A Novel Approach to Detect a Pathological Process
Source: PLoS One. 2010 Nov 4;5(11):e13813. doi: 10.1371/journal.pone.0013813 (PMC2973952; doi:10.1371/journal.pone.0013813)
Supplement: Table S4 — Volatile compounds with significant increase in frequency distribution from frozen skin (M) melanoma, S-skin. (*-N/A). (0.01 MB DOCX) [file pone.0013813.s005.docx]

| **No** | **CAS** | **COMPOUND** | **M** | **S** | **p-value** | **odds ratio** |
| --- | --- | --- | --- | --- | --- | --- |
| 1 | 63523-85-3 | 1-Methyl-bis(1,2,4)-triazole-5,1' | 5/21 | 15/20 | 0.0012 | 9.6 |
| 2 | 23676-09-7 | Benzoic acid, 4-ethoxy-, ethyl ester | 2/21 | 13/20 | 0.0003 | 17.6 |
| 3 | 5989-27-5 | D-Limonene | 2/21 | 9/20 | 0.0114 | 7.7 |
| 4 | 112-31-2 | Decanal | 1/21 | 6/20 | 0.034 | 8.6 |
| 5 | 100-41-4 | Ethylbenzene | 10/21 | 17/20 | 0.012 | 6.2 |
| 6 | 111-71-7 | Heptanal | 1/21 | 13/20 | <0.0001 | 37 |
| 7 | 66-25-1 | Hexanal | 3/21 | 12/20 | 0.003 | 9 |
| 8 | 124-19-6 | Nonanal | 8/21 | 20/20 | <0.0001 | * |
| 9 | 124-13-0 | Octanal | 1/21 | 9/20 | 0.0031 | 16.3 |
| *10* | *106-42-3* | P-Xylene | *8/21* | *15/20* | *0.018* | *4.8* |
