# Supplementary material for: Differential Volatile Signatures from Skin, Naevi and Melanoma: A Novel Approach to Detect a Pathological Process
Source: PLoS One. 2010 Nov 4;5(11):e13813. doi: 10.1371/journal.pone.0013813 (PMC2973952; doi:10.1371/journal.pone.0013813)
Supplement: Table S5 — Common compounds present at ≥40% of control air blank samples. (0.01 MB DOCX) [file pone.0013813.s006.docx]

| **CAS** | **COMPOUND** |
| --- | --- |
| **84-69-5** | 1,2-Benzenedicarboxylic acid, bis(2-methylpropyl) ester |
| **104-76-7** | 1-Hexanol, 2-ethyl- |
| **15892-23-6** | 2-Butanol |
| **621-82-9** | 2-Propenoic acid, 3-phenyl |
| **640-19-7** | Acetamide, 2 fluoro- |
| **64-19-7** | Acetic acid |
| **91-08-7** | Benzene, 1,3-diisocyanato-2-methyl- |
| **108-38-3** | Benzene, 1,3-dimethyl- |
| **584-84-9** | Benzene, 2,4-diisocyanato-1-methyl- |
| **117-81-7** | Bis(2-ethylhexyl) phthalate |
| **540-18-1** | Butanoic acid, pentylester |
| **79-92-5** | Camphene |
| **555-10-2** | β-Phellandrene |
| **112-31-2** | Decanal |
| **84-74-2** | Dibutyl phthalate |
| **84-66-2** | Diethyl phthalate |
| **112-40-3** | Dodecane |
| **64-17-5** | Ethanol |
| **111-90-0** | Ethanol,2-(2-ethoxyethoxy)- |
| **100-41-4** | Ethylbenzene |
| **75-21-8** | Ethylene oxide |
| **64-18-6** | Formic acid |
| **60-34-4** | Hydrazine, methyl |
| **67-63-0** | Isopropyl Alcohol |
| **138-86-3** | Limonene |
| **124-19-6** | Nonanal |
| **111-84-2** | Nonane |
| **111-65-9** | Octane |
| **108-83-3** | Toluene |
| **629-50-5** | Tridecane |
| **1120-21-4** | Undecane |
| **95-47-6** | o-Xylene |
| **106-42-3** | p-Xylene |
